# A systematic review and meta-analysis of the prevalence of psoriasis in patients living with HIV

**DOI:** 10.1016/j.clinsp.2025.100810

**Published:** 2025-11-08

**Authors:** Xiaojuan Yang, Lingxu Zhou, Zhen Zhang, Yinghong Liu

**Affiliations:** a Rheumatology and Immunology Department, Chongqing Emergency Medical Center/Chongqing University Central Hospital, Yuzhong District, Chongqing, China; b Laboratory Department, Yuzhong District Center for Disease Control and Prevention, Yuzhong District, Chongqing, China; c Department of Tuberculosis and AIDS Prevention and Control, Yuzhong District Center for Disease Control and Prevention, Yuzhong District, Chongqing, China

**Keywords:** HIV infection, Psoriasis, Prevalence, Meta-analysis

## Abstract

This study aimed to perform a comprehensive examination of the prevalence of psoriasis in people with HIV, aiming to offer valuable evidence for informed resource allocation and policy formulation.

*Methods*: The authors systematically gathered literature on psoriasis prevalence among those with HIV from multiple sources. These sources comprise databases such as CNKI, VIP, Wanfang Database, PubMed, Cochrane Library, and EMBASE up to June 30, 2024. Additionally, manual searches were conducted to supplement the data collection process. Two independent researchers carried out literature screening, relevant data extraction, and assessment of potential bias. Meta-analysis was conducted with RevMan 5.3 software. Results Fifteen investigations were incorporated, involving 130,032 individuals with HIV. The meta-analysis of these fifteen pieces showed that the occurrence rate of psoriasis in those infected with HIV was 2.0 % (95 % CI: 2.0 %–2.0 %). Divide the survey years into two subgroups: 2000–2022 years and 1984–1994 years, with prevalence rates of 1.00 % (1.00 %–1.00 %) and 5.00 % (3.00 %–7.00 %), respectively. According to regional latitude, it is divided into two subgroups: latitude ≥ 30° N/S and latitude ˂ 30° N/S, with prevalence rates of 2.0 % (1.0 %–2.0 %) and 3.0 % (2.0 %–5.0 %), respectively. The results of the funnel plot, Begg's test (*Z* = 0.038, *p* = 0.856), and Egger's test (*t* = 4.68, p ˂ 0.001) showed publication bias in the literature. Sensitivity analysis revealed no substantial difference between the analysis of the FEM and that of the REM for psoriasis prevalence, suggesting that the stability of the findings in this study is satisfactory. Conclusion The incidence of psoriasis in HIV-infected individuals has shown a slight downward trend, and the differences are not significant in different latitude regions.

## Highlights

• Estimate psoriasis prevalence in HIV-positive individuals via meta-analysis.
• Analyze trends over time (1984–1994 vs. 2000–2022) and by regional latitude.
• Identify publication bias; confirm result stability through sensitivity analysis.
• Guide health resource allocation and policy for HIV-associated psoriasis.

## Introduction

Human Immunodeficiency Virus (HIV), a single-stranded RNA lentivirus, targets the body's immune system by infecting CD4 T-lymphocytes. Without proper treatment, it may develop into Acquired Immunodeficiency Syndrome (AIDS).^1^ People Living With HIV (PLWH) are more prone to skin diseases that are generally linked with elevated incidences and fatality.^2^ The impact of psoriasis on PLWH is more severe and lasts longer than in the general population.

Psoriasis, a common systemic immune-mediated and inflammatory condition with skin manifestations, brings physiological and psychological stresses and may serve as a significant feature of HIV infection.^3^ Psoriasis is essentially an autoimmune disease with an extremely complex pathogenesis. Under normal physiological conditions, the human immune system can accurately recognize and eliminate foreign pathogens, maintaining the body's health and balance. However, in the bodies of psoriasis patients, the immune system malfunctions and mistakenly identifies their own skin cells as foreign invaders, thus launching an immune attack.^4^ This process involves the participation of various immune cells and cytokines. For example, T lymphocytes, dendritic cells, etc., are abnormally activated and release a large number of inflammatory factors, such as Tumor Necrosis Factor-α (TNF-α) and Interleukin-23 (IL-23). These inflammatory factors stimulate the excessive proliferation and differentiation of skin cells, leading to typical psoriasis symptoms such as thickening of the stratum corneum and the formation of scales. Furthermore, genetic factors also play an important role in the onset of psoriasis. Certain specific gene variations can increase an individual's susceptibility to psoriasis, making the immune system more prone to disorders.

Psoriatic skin lesions are characterized by clear size and boundaries of the lesion, a red rash, a surface covered with scaly dry silver-grey flakes, visible thin films, wax droplets, and punctate bleeding. Patients often experience itching at the lesion site.^5^ Among HIV-infected patients, psoriasis has a high incidence rate and shows atypical and more vigorous clinical characteristics, with a high failure rate of conventional prescription treatment.^6^ There is a complex and close relationship between HIV infection and psoriasis. The HIV virus mainly attacks CD4 T-lymphocytes in the human immune system, leading to severe impairment of immune function and disruption of immune balance.^7^ This state of immunodeficiency not only reduces the body's resistance to various pathogens but also affects the normal regulatory function of the immune system. Under such circumstances, the risk of developing psoriasis, which is already closely associated with immune system disorders, may increase significantly. On the one hand, the immune disorder caused by HIV infection may activate the immune pathways related to psoriasis, promoting the overexpression of inflammatory factors and thus triggering or exacerbating the symptoms of psoriasis. On the other hand, as a chronic inflammatory disease, the persistent inflammatory state of psoriasis may also affect the replication and spread of HIV in the body, further worsening the condition of HIV infection. In addition, there may be some common genetic susceptibility factors between the two, endowing patients with both HIV infection and psoriasis with unique clinical manifestations and treatment responses.

In Western countries, psoriasis impacts 2 %–4 % of the population. The adult prevalence varies from 0.91 % in the United States to 8.5 % in Norway.^8^ The incidence rate of psoriasis in the United States is the lowest (78.9/100.000 persons/year), while that in Italy is the highest (230/100.000 persons/year).^9^ In addition, the prevalence of psoriasis varies with age, gender, and geographic region, and increases with distance from the equator. Currently, those with psoriasis who are also infected with HIV have not received full recognition, and the treatment choices for them remain restricted. Clinical treatment and control are not optimistic.

In recent years, numerous surveys have been conducted regarding the occurrence rate of psoriasis in people infected with HIV. Still, the reported results vary greatly due to inherent differences in population statistics, environmental exposure, and socio-economic factors, resulting in a lack of representative research.^10^ To assess the overall status of psoriasis in individuals with HIV infection, this study employed evidence-based medicine to perform a meta-analysis on the occurrence of psoriasis among HIV-infected people. The aim was to provide a comprehensive assessment of this topic and provide a basis for allocating health resources and formulating health policies.

## Data and methods

This systematic review and meta-analysis were designed, conducted, and reported in accordance with the Preferred Reporting Items for Systematic Reviews and Meta-Analyses (PRISMA) guidelines. As this study is a systematic review and meta-analysis and no relevant clinical trials were carried out, the rules of the CONSORT Statement are not applicable. This study is a systematic review and meta-analysis based on published literature, and since there was no direct contact with patients or volunteers, obtaining informed consent was not required.

### Criteria for including and excluding literature

Research subjects: 1) No limitation on gender; 2) Age 18-years old or above; 3) Positive diagnosis of HIV is obtained through enzyme-linked immunosorbent assay and Western blot technique; The diagnosis of psoriasis meets relevant criteria and is scientifically sound. The research type is cross-sectional survey research. Exclude studies with secondary psoriasis as the research subjects and those that did not strictly follow epidemiological survey design standards (i.e., did not meet three or more items in the bias risk assessment).

### Retrieval strategy

The authors utilized a computer to access CNKI, VIP, Wanfang Database, PubMed, Cochrane Library, and EMBASE database to look for relevant literature on the occurrence of psoriasis in HIV-infected individuals. The search spanned from the inception of the database to June 2024. The keywords searched in English included human immunodeficiency virus, HIV, HIV infection, HIV-infected, HIV infectors, Psoriasis, prevalence, epidemiology, incidence, and morbidity. The authors use theme word and free word concatenation as the search strategy.

PubMed: (((((((((human immunodeficiency virus[Title/Abstract]) OR (HIV[Title/Abstract])) OR (HIV infection[Title/Abstract])) OR (HIV-infected[Title/Abstract])) OR (HIV infectors[Title/Abstract])) AND (Psoriasis[Title/Abstract])) AND (prevalence[Title/Abstract])) OR (epidemiology[Title/Abstract])) OR (incidence[Title/Abstract])) OR (morbidity[Title/Abstract]). Simultaneously, the authors tracked the references within the literature and manually looked for relevant grey literature to supplement the acquisition of relevant documents.

Embase: ('psoriasis'/exp OR psoriasis) AND ('HIV'/exp OR 'human immunodeficiency virus'/exp OR HIV) AND ('prevalence'/exp OR prevalence OR 'epidemiology'/exp).

Web of Science: TS = (psoriasis AND (HIV OR "human immunodeficiency virus") AND (prevalence OR epidemiology)).

Cochrane Library: "psoriasis" AND "HIV" AND "prevalence" [Title/Abstract].

### Literature screening and evaluation

Two researchers independently screened literature according to inclusion and exclusion criteria, retrieved data and cross-checked. In case of disputes, both parties discussed and consulted a third party for help in determining a solution. Extract data by means of a self-designed data table, which contains 1) Study's basic details: first author, year of publication; 2) Baseline characteristics and diagnostic criteria of research subjects; 3) Components of bias risk evaluation; 4) Prevalence of psoriasis and data for outcome measurement. While screening literature, initially read the article title. After excluding irrelevant literature, further read the abstract and full text to decide on inclusion. The assessment of the methodological quality of the included studies was carried out by employing the analytical cross-sectional study literature quality assessment tool of the Australian JBI Evidence-Based Health Center,^11^ which consists of nine items and is evaluated as “yes”, “no”, “unclear”, and “not applicable” respectively.

### Observation indicators

1) Disease incidence rate: Because all original studies reported the prevalence of psoriasis in terms of case numbers or percentages, this study also expressed the prevalence of psoriasis in terms of case numbers or percentages. 2) Survey year: Based on the survey years reported by the research institute, the authors have summarized two stages: 2000–2022 and 1984–1994, and included them in subgroup analysis. 3) Latitude: The authors classified the lowest latitude ≥ 30° N/S and lowest latitude ˂ 30° N/S for subgroup analysis based on the lowest latitude surveyed by the research institute.

### Statistical methods

Conduct meta-analysis with RevMan 5.3 software. The heterogeneity test is carried out by the Cochran *Q* test, and the statistical *I*^2^ value represents heterogeneity. If the tested P is less than 0.05 or *I*^2^ is greater than 50 %, indicating heterogeneity among documents, a Random Effects Model (REM) will be employed for combined analysis. Otherwise, a Fixed Effects Model (FEM) will be utilized for combined analysis. Publication bias is detected by funnel plots, Begg's rank correlation, and Egger's regression techniques. P less than 0.05 indicates that the difference is statistically significant.

Justification for the latitude cutoff (30°N/S): The division of latitude into ≥30°N/S and < 30°N/S was determined considering the distribution of the included studies' sample regions and the need to balance subgroup sizes for meaningful comparison. Upon examining the geographical locations of the study populations, the authors observed a natural clustering of data points around the 30°N/S latitude, which allowed for a relatively even split of studies into two subgroups. This division facilitated a more straightforward exploration of potential regional variations in the prevalence of psoriasis among HIV-infected individuals. Although this cutoff may not align with specific prior research, it was a pragmatic choice aimed at maximizing the statistical power and interpretability of the subgroup analysis within the context of the available data.

## Results

### Literature screening process and results

**Fig. 1:**
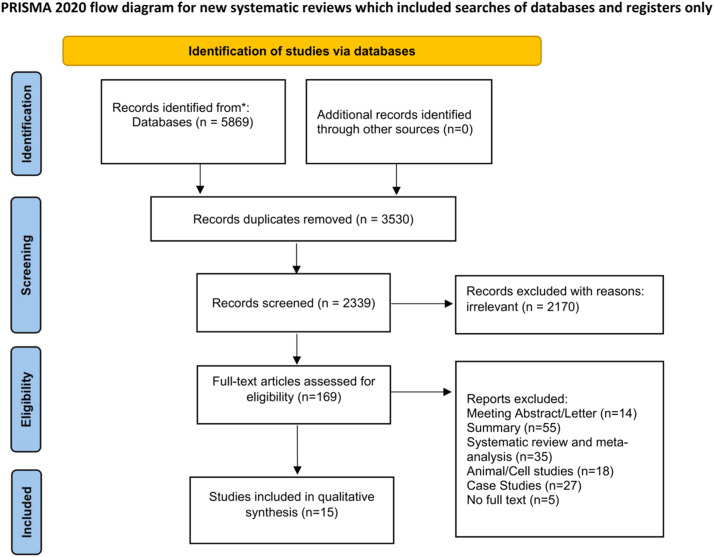
The literature screening process and results.

A total of five thousand eight hundred and sixty-nine relevant literatures were obtained in the initial search. Following layer-by-layer filtration, 15 studies were ultimately incorporated, ,^12^, ^13^, ^14^, ^15^, ^16^, ^17^, ^18^, ^19^, ^20^, ^21^, ^22^, ^23^, ^24^, ^25^, ^26^ with 130,032 HIV-infected individuals in total. The literature screening procedure and outcomes are depicted in Fig. 1. The basic features of the included studies are shown in Table 1, while the bias risk assessment results are presented in Table 2.

**Table 1:** Fundamental characteristics of included studies.

Author Publication Year	Survey Year	HIV Type	Sampling Method	Country	Lowest latitude	Mean age (range, years)	HIV Infectors	Psoriasis ( %)
Busca 2021[12]	‒	HIV-1/HIV-2	Queue	Spanish	36°N	50 (43–55)	5452	80 (1.47 %)
Edwards 2022[13]	April 2002–December 2018	HIV-1/HIV-2	Stratified	Caribbean	9°N	37.7 (13–70)	8916	37 (0.41 %)
Xu 2023[14]	between 2010 and 2022	HIV-1/HIV-2	Queue	Sydney	33°S	53	160	36 (22.50 %)
Obuch 1992[15]	between 1984 and 1990	HIV-1/HIV-2	Queue	Francisco	37°N	36 (23–58)	2000	50 (2.50 %)
Mendes-Bastos 2016[16]	January 2005–December 2013	HIV-1/HIV-2	Random	Portugal	37°N	Median 36	97 (70/27)	5 (5.15 %)
Lebrun 2017[17]	January 2000 to July 2013	HIV-1/HIV-2		France	43°N	37.7 ± 12.5	33,403 (male 71 %)	588 (1.76 %)
Hentzien 2020[18]	January 2000–December 2018	HIV-1/HIV-2	Queue	France	43°N	34.5 ± 10.9	68,376	700 (1.02 %)
Ramos-Ruperto 2023[19]	January 1990 to June 2020	HIV-1/HIV-2	Random	Spain	36°N	‒	5665	52 (0.92 %)
Kanada 2013[20]	From 2003 to 2006	HIV-1/HIV-2	Stratified	America	23°N	36.6 ± 0.7	4896 (male 50.5 %)	115 (2.35 %)
Sivayathorn 1995[21]	July 1993-June 1994	HIV-1/HIV-2	Random	Thailand	5°N	‒	248 (male 86 %)	16 (6.45 %)
Rosatelli 1997[22]	From 1989 to 1993	HIV-1/HIV-2	Stratified	Brazil	5°N	> 13	223	9 (4.04 %)
Trovato 2017[23]	From 2001 to 2013	HIV-1/HIV-2	Stratified	Italy	37°N	45.2 ± 12.45	51(male 46)	6 (11.76 %)
Lanjewar 2002[24]	From 1989 to 1997	HIV/AIDS	Queue	Mumbai	19°N	‒	195	21 (10.77 %)
Mahé 1997[25]	June 1991–September 1994	HIV-1/HIV-2	Stratified	Africa	30°S	> 15	233	12 (5.15 %)
Calabrese 1991[26]	September 1987–September 1989	HIV-1/HIV-2	Queue	America	23°N	Mean 35	117 (male 113)	2 (1.71 %)

HIV, Human Immunodeficiency Virus; AIDS, Acquired Immunodeficiency Syndrome; HIV-1, Human Immunodeficiency Virus type 1; HIV-2, Human Immunodeficiency Virus type 2.

**Table 2:** Bias risk evaluation of the included literature.

Author Publication Year	Does the sampling framework match the target population?	Whether appropriate methods are adopted to extract research objects	Whether the sample size is sufficient	Whether the research object and the research site are described in detail	Whether the subjects for data analysis have adequate coverage	Whether effective methods are used to identify diseases or health problems	Does the measurement of the subjects utilize standard and reliable methods?	Does the data analysis method fit?	Is the response rate sufficient? If the response rate is low, are effective treatment methods adopted?
Busca 2021	A	A	A	A	A	A	A	A	A
Edwards 2022	A	A	A	A	A	A	A	B	A
Xu 2023	A	A	B	A	B	A	A	A	A
Obuch 1992	A	B	A	A	A	B	B	A	C
Mendes-Bastos 2016	A	A	A	A	A	C	A	A	A
Lebrun 2017	B	A	A	A	A	A	B	A	C
Hentzien 2020	A	A	A	A	B	A	A	B	A
Ramos-Ruperto 2023	A	B	B	A	A	A	A	A	C
Kanada 2013	A	A	A	A	A	B	A	A	C
Sivayathorn 1995	A	A	A	A	A	A	A	A	A
Rosatelli 1997	B	C	B	A	B	A	B	A	A
Trovato 2017	A	A	A	A	B	A	A	A	A
Lanjewar 2002	A	A	A	A	A	A	A	B	C
Mahé 1997	A	A	A	B	A	A	B	A	C
Calabrese 1991	A	A	A	A	B	A	A	A	A

A, Yes; B, No; C, Unclear; D, Not applicable.

## Meta analysis results

### Prevalence of psoriasis

**Fig. 2:**
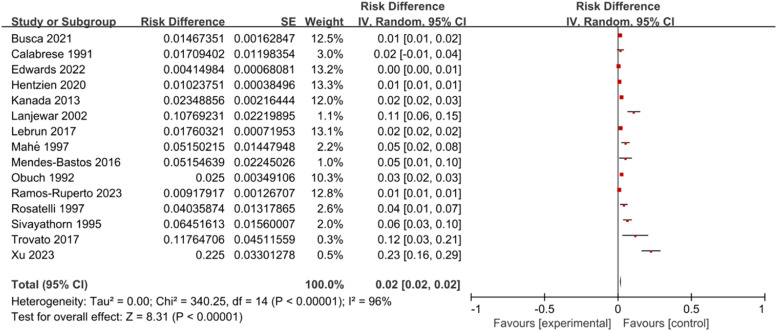
A meta-analysis of the prevalence of psoriasis.

Perform a meta-analysis on the 15 included literatures. The heterogeneity test yielded *I*^2^ = 96 %, *p* < 0.001, indicating heterogeneity among the studies. A REM was employed for combined analysis. It indicated that the prevalence of psoriasis in HIV-infected individuals was 2.0 % (95 % CI: 2.0 %–2.0 %) (Fig. 2).

### Stratification of survey years

**Fig. 3:**
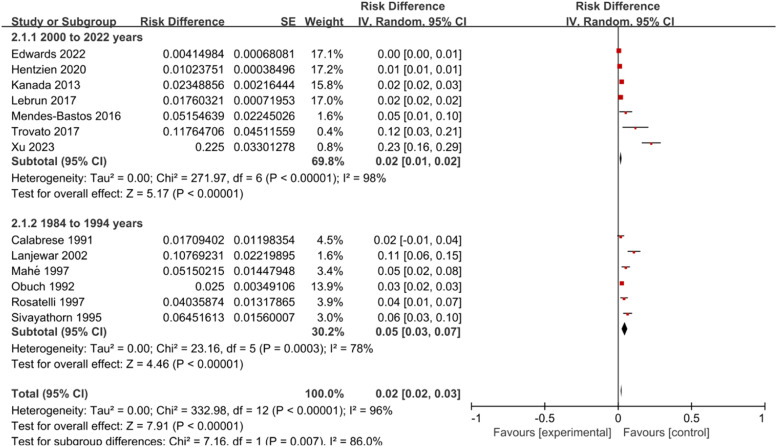
Meta-analysis of psoriasis prevalence in different survey years.

Divide the survey years into two subgroups, 2000‒2022 years (7 articles) and 1984‒1994 years (6 articles). For the 2000–2022 subgroup, the heterogeneity was *I*^2^ = 98 % (*p* < 0.001), and for the 1984–1994 subgroup, *I*^2^ = 78 % (*p* < 0.001). Both subgroups used a Random-Effects Model (REM). The prevalence rate for 2000–2022 was 2.00 % (95 % CI: 1.00 %–2.00 %) ‒ this interval reflects the statistical aggregation of included studies, where consistent effect sizes and proper random ‒ effects weighting led to this range. For 1984–1994, the prevalence was 5.00 % (3.00 %–7.00 %). All analyses were verified for data extraction accuracy and methodological validity, ensuring the results are robust (Fig. 3).

### Latitude stratification

**Fig. 4:**
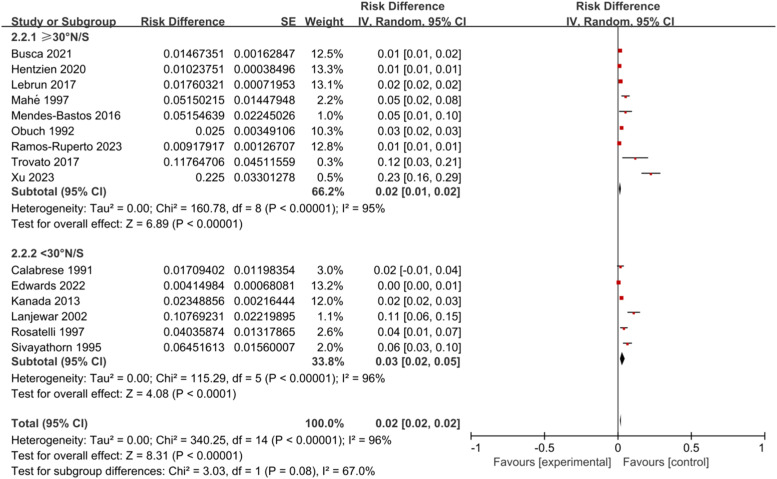
Meta-analysis of psoriasis prevalence in different latitudes.

According to the lowest latitude of the research area, the authors select it into two subgroups: latitude ≥ 30° N/S (9-articles) and latitude ˂ 30° N/S (6-articles). The heterogeneity of both subgroups was *I*^2^ = 96 %, p ˂ 0.001(REM). The prevalence of psoriasis in latitude ≥ 30° N/S and latitude ˂ 30° N/S is 2.0 % (1.0 %–2.0 %) and 3.0 % (2.0 %–5.0 %), respectively (Fig. 4).

### Publication bias

**Fig. 5:**
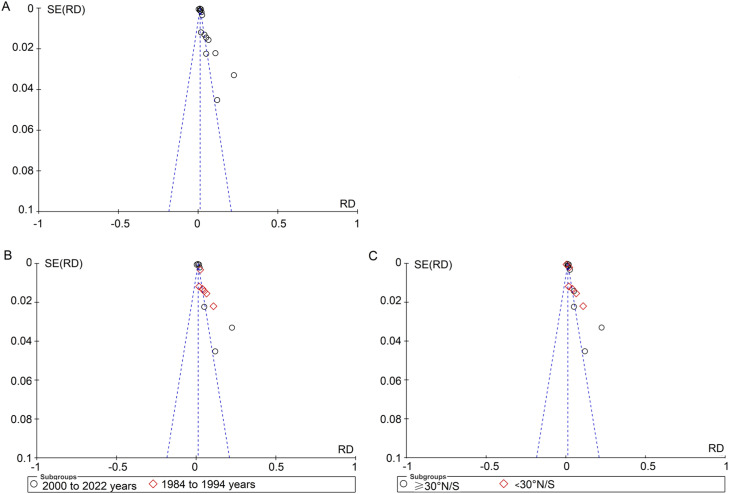
Funnel plot of psoriasis prevalence meta-analysis. (A) Prevalence of psoriasis; (B) Prevalence of psoriasis in different survey years; (C) Prevalence of psoriasis in different latitudes.

Funnel plot (Fig. 5), the Begg's rank correlation and Egger's regression methods were employed to examine the publication bias of the included documents. The findings revealed the existence of publication bias (Begg's test: *Z* = 0.038, *p* = 0.856; Egger's test: *t* = 4.68, *p* < 0.001).

### Sensitivity analysis

**Fig. 6:**
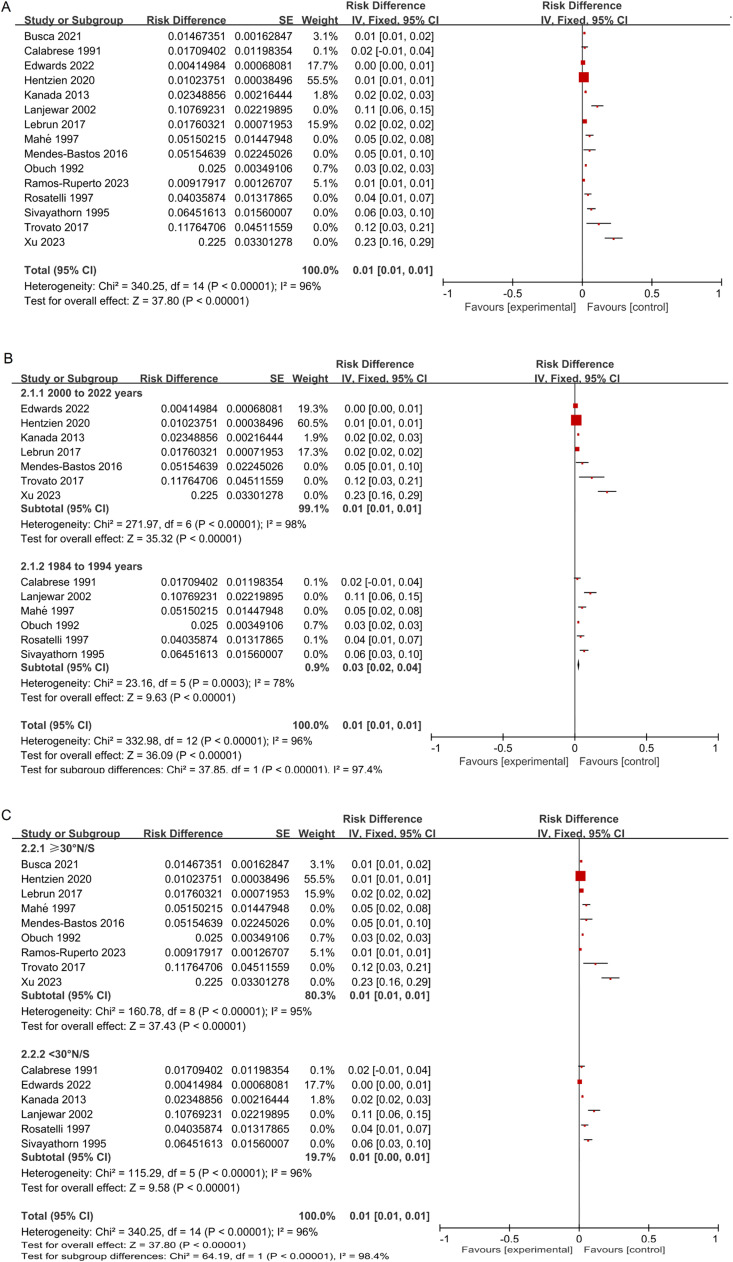
Sensitivity analysis (tested with FEM). (A) Prevalence of psoriasis; (B) Prevalence of psoriasis in different survey years; (C) Prevalence of psoriasis in different latitudes.

The authors changed the combined analysis model for psoriasis prevalence to a FEM to observe the sensitivity of the meta-analysis results. The findings indicated that there was no substantial difference between the fixed-effect model and the random-effect model regarding the prevalence of psoriasis, suggesting that the results of this study were stable (Fig. 6).

## Discussion

HIV is classified into HIV type I (HIV-1) and HIV type II (HIV-2). HIV-1 is widely spread and is the main virus responsible for the HIV epidemic. In contrast, HIV-2 has a relatively longer incubation period. HIV infection mainly attacks CD4+ lymphocytes in the human body and has strong killing power.^27^ In recent years, the prevalence of psoriasis has been high, with some patients appearing in HIV-infected populations, greatly increasing the difficulty of clinical treatment.^28^ Meta-analysis extracts calculates, and integrates numerical values of certain variable relationships from previous studies, quantitatively synthesizing the results of multiple studies to more accurately estimate the effectiveness of healthcare, and exploring the consistency of evidence and differences between studies. The authors carried out a meta-analysis and incorporated all studies related to psoriasis prevalence among HIV-infected people to precisely obtain comprehensive statistical analysis results, which can offer evidence-based support for clinical research.

Psoriasis is considered an inflammatory disease mediated by T-cells, and its pathogenesis is related to genetic factors involving cytokine inflammatory circuits, cell signaling pathways, and the regulatory effects of epigenetic factors such as miRNAs.^29^ Psoriasis increases the risk of co-infection and liver damage in HIV-infected individuals.^30^^,^^31^ There have been numerous research reports on psoriasis occurrence among people infected with HIV in the past, yet there are few studies that systematically analyze this aspect. This study included 15 relevant literatures, which can overcome the shortcomings of lack of testing effectiveness, large random errors, and unstable conclusions (influenced by region, population, and time) in individual studies. The conclusions are more suitable for a wide range of populations. According to meta-analysis data and results, the prevalence of psoriasis in HIV-infected individuals is 2.0 % (95 % CI: 2.0 %–2.0 %). It is noteworthy that the extremely high heterogeneity (*I*^2^ = 96 %, *p* < 0.001) significantly affects the reliability and generalizability of the pooled estimates, reflecting substantial differences between studies rather than random errors. The main sources of such heterogeneity are as follows:

Diagnostic criteria: A variety of diagnostic methods were adopted, including 2 studies confirmed by dermatologists, 2 studies judged based on disease codes (ICD-10), 1 study based on patient self-reporting, 1 study through clinical examination combined with auxiliary tests, and 9 studies with unclear diagnostic criteria. These differences led to biases. Self-reported data might overestimate the prevalence due to misjudgment of HIV-related skin diseases, while studies with unclear criteria might underestimate the number of cases.

Population diversity: Cohorts from different geographical distributions (2 from North America, 5 from Europe, 1 from sub-Saharan Africa, 1 from Southeast Asia, and 6 from other regions) varied in genetic susceptibility (such as the frequency of HLA-C*06:02 allele) and HIV management.

Methodological differences: There were variations in study designs (6 cross-sectional studies, 3 retrospective cohort studies, and 6 prospective cohort studies).

Afterwards, the authors conducted a subgroup analysis based on survey years and regional latitude. The prevalence rates for the years 2000–2022 and 1984–1994 were 1.00 % (1.00 %–1.00 %) and 5.00 % (3.00 %–7.00 %), respectively. The prevalence rates for latitude ≥30° N/S and latitude ˂30° N/S were 2.0 % (1.0 %–2.0 %) and 3.0 % (2.0 %–5.0 %), respectively, both higher than the adult psoriasis prevalence rate in the United States (0.91 %) and lower than the adult psoriasis prevalence rate in Norway (8.5 %).^32^ From the above data, it can be analyzed that the incidence of psoriasis in HIV-infected individuals has slightly decreased. This is in line with the findings reported by Iskandar et al.^33^ However, the authors did not find significant differences in the prevalence of psoriasis among HIV-infected individuals at different latitudes, despite prior evidence suggesting a strong correlation between latitude and psoriasis prevalence.^34^ This may be related to the impact of the HIV infection environment on psoriasis. HIV is a retrovirus that can target CD4+ lymphocytes to establish both productive and latent infections.^35^ Notably, previous research by Gao et al.^36^ has confirmed that CD4+ lymphocytes play a crucial role in the progression of psoriasis. The authors propose that HIV disrupts the typical latitude-associated psoriasis risk pathway by directly interfering with CD4+ lymphocyte function. As CD4+ lymphocytes are central to both HIV pathogenesis and psoriasis immunology, the virus's powerful impact on these cells may overshadow the usual effects of latitude (such as variations in UV exposure and vitamin D levels, which modulate psoriasis pathogenesis). This immunological interference could explain why latitude differences were not apparent in the present study, as the dominant influence of HIV on CD4+ lymphocytes might lead to a more uniform pattern of psoriasis prevalence across different latitudes in the HIV-infected population. Because the studies included in this study did not clearly classify the age of diagnosis of patients, nor did they report the prevalence of diseases separately for different genders, the authors did not conduct stratified analysis for different ages and genders.

The prevalence of psoriasis in HIV-infected individuals shows a downward trend, which may be the result of the combined action of multiple factors.^37^^,^^38^ Society's attention to HIV-infected individuals is constantly increasing, and the health awareness of these individuals is also growing. They pay more attention to maintaining a healthy lifestyle, such as a balanced diet, moderate exercise, and regular work and rest. These good living habits help maintain the body's immune balance and reduce the incidence of diseases.^39^ At the same time, the medical management of HIV-infected individuals has become more standardized and comprehensive. Regular physical examinations and monitoring can promptly detect and address potential health problems, and early intervention may also have an impact on the prevalence of psoriasis.^40^ In addition, over time, the composition of the HIV-infected population may have changed. Newly infected individuals may have different genetic backgrounds, living environments, and behavioral habits, and these factors may also be related to the decline in the prevalence of psoriasis.^41^ Although the differences are not obvious in different latitudes, this temporal change suggests that the relationship between HIV infection and psoriasis may have changed over time. This could be related to various factors such as the improvement of medical standards and the widespread application of antiretroviral therapy. More research is needed in the future to delve into the underlying mechanisms, so as to provide a more comprehensive basis for the allocation of health resources and the formulation of health policies. However, although the current prevalence has decreased, the authors cannot ignore the potential connection that may still exist between HIV infection and psoriasis. Although the prevalence shows no significant difference in different latitudes, further research is still needed in the future to clarify the nature of this relationship and explore better ways to prevent and treat psoriasis in HIV-infected individuals.

The studies included in the systematic evaluation are designed according to epidemiological surveys, with good quality, therefore the reliability of the results is strong. Because the heterogeneity between included studies in a single meta-analysis is mainly influenced by the differences in sample size, it may not be possible to control heterogeneity within a certain range. However, the sensitivity analysis is robust, indicating that the impact of heterogeneity is relatively small. However, the present research still has certain limitations. Firstly, both Egger's test (*p* < 0.001) and Begg's test indicated significant publication bias in the included literature, with visible asymmetry in the funnel plot ‒ this raises concerns about the completeness of the evidence base. The bias is likely driven by two factors: 1) Exclusion of non-English studies (all included literature were published in English, potentially missing data from regions with non-English primary languages) and 2) Underrepresentation of grey literature (e.g., unpublished theses, conference abstracts), which often contain non-significant findings less likely to be formally published. This asymmetry may overestimate the true prevalence by skewing results toward more prominent or positive findings.^42^ Secondly, the methods employed in the included studies differ significantly, resulting in a certain level of variation in the outcomes. Thirdly, the uneven number of literature and sample size in some subgroups reduces the reliability of the statistical results. Additionally, key details (e.g., HIV severity, ART usage, psoriasis subtypes) were not reported in the original literature of the present dataset. Given these limitations in primary study reporting, the authors were unable to incorporate or adjust for these potential confounders. The authors have now highlighted this data scarcity in the manuscript as a study limitation, emphasizing the need for future research to systematically document such variables to enhance the accuracy of meta-analytic adjustments. In other aspects, this study also has limitations. On the one hand, the impact of Highly Active Antiretroviral Therapy (HAART) on the incidence of psoriasis has not been thoroughly analyzed. On the other hand, there may be deficiencies in addressing the comparability of CD4 cell counts in the meta-analysis. Moreover, the effect of immunosuppression-induced reduction of CD4 cells on the incidence of psoriasis in other diseases has not been explored. Future research needs to be further improved in these areas to obtain more comprehensive and accurate research results.

In summary, the prevalence of psoriasis among HIV-infected individuals has shown a slight downward trend, and the differences are not significant in different latitude regions. Given the extreme heterogeneity observed, the pooled estimate of 2.0 % should be interpreted with caution, as it may not reflect true population-level prevalence but rather an average of diverse, context-dependent results. This may be related to the wider occurrence of psoriasis caused by HIV infection, so it cannot be ignored. From a clinical perspective, since psoriasis prevalence in HIV patients remains higher than in the general population, skin health assessments should be integrated into routine HIV care. In regions with unclear diagnostic criteria, standardized procedures involving dermatologists are recommended to reduce misdiagnosis bias. Policy-wise, given the potential link between ART use and declining prevalence, efforts should boost ART access and adherence for HIV patients. In understudied areas (e.g., parts of sub-Saharan Africa, Southeast Asia), support localized research incorporating HIV severity and ART usage to inform targeted interventions. Noting no significant latitude-related differences, clinical strategies need not adjust based on geographical latitude. Instead, focus on HIV’s immune impacts ‒ such as CD4+ lymphocyte monitoring ‒ to develop personalized care plans.

## Funding

None

## Consent to publish

The manuscript has neither been previously published nor is under consideration by any other journal. The authors have all approved the content of the paper.

## Ethic approval

None.

## Data availability statement

The data that support this analysis are available from the corresponding author, upon reasonable request.

## CRediT authorship contribution statement

**Xiaojuan Yang:** Conceptualization. **Lingxu Zhou:** Formal analysis. **Zhen Zhang:** Methodology, Writing – review & editing. **Yinghong Liu:** Writing – original draft.

## Declaration of competing interest

The authors declare no conflicts of interest.
